# Comparison of root tolerance to drought and aphid (*Myzus persicae* Sulzer) resistance among different potato (*Solanum tuberosum* L.) cultivars

**DOI:** 10.1038/s41598-020-79766-1

**Published:** 2021-01-12

**Authors:** Peter Quandahor, Yuping Gou, Chunyan Lin, Jeffrey A. Coulter, Changzhong Liu

**Affiliations:** 1 College of Plant Protection, Gansu Agricultural University, Lanzhou, No. 1 Yingmen Village, Anning District, Lanzhou, 730070 People’s Republic of China; 2 Department of Agronomy and Plant Genetics, University of Minnesota, St. Paul, Minnesota, 55108 USA

**Keywords:** Ecology, Physiology, Plant sciences

## Abstract

This study was conducted to determine the root system architecture and biochemical responses of three potato (*Solanum tuberosum* L.) cultivars to drought and aphid (*Myzus persicae* Sulzer) infestation under greenhouse conditions. A factorial experiment comprising three potato cultivars (Qingshu 9, Longshu 3, and Atlantic), two levels of water (Well watered and drought) application and aphid infestation (Aphids and no aphids) was conducted. The results show that drought stress and aphid infestation significantly increased the root-projected area, root surface area, number of root tips, and number of root forks of all cultivars, relative to their corresponding control plants. The least root projected area, root surface area, number of root tips, and number of root forks occurred on DXY under both drought and aphid infestation. Nevertheless, the greatest root projected area, root surface area, number of root tips and number of root forks occurred on QS9 plants. Moreover, increased SOD, CAT, and POD activities were observed across all cultivars, under drought and aphid stress. The highest SOD, POD, and CAT activities occurred in QS9; under drought and aphid stress, while the least SOD, POD, and CAT activities was observed in DXY. The Atlantic cultivar, which possesses a root system sensitive to water deficit, demonstrated greater resistance to aphid infestation under well-watered and drought-stressed conditions. Conversely, Qingshu 9, which possesses a root system tolerant to water deficit, was highly susceptible to aphids. This study shows that the root architectural and biochemical traits that enhance potato tolerance to drought do not necessarily correlate to a plant’s tolerance to aphids.

## Introduction

Plant roots are the main organs for transporting diverse resources from the soil, and thereby control plant efficiency^1^. The mechanism by which plant roots obtain water and nutrients from soil is complex and it involves several abiotic and biotic interactions^2^. Plants may develop deep or fibrous root systems in order to obtain available soil moisture for survival^3^. The increase in root length and proliferation of roots for drought tolerance has been reported in several crops, including *Oryza sativa*^4^, *Zea mays*^5^, *Hordeum vulgare*^6^, *Triticum aestivum*^7^, *Brassica napus*^8^, and *Glycine max*^9^*.* Root diameter and number of root forks that determines root conductivity haves also been found to improve drought tolerance in legumes^10^. Smaller root diameter efficiently increases hydraulic conductance by increasing the root surface area that can be used for water uptake^11^. Hence, a decrease in root diameter has been suggested as a trait for improving plant transmission of resources under stress conditions^7^. Root crossing (root branching) governs the bearing of vertical and horizontal distribution of roots in the soil, and is predicted as a vital trait for drought tolerance in *Sorghum bicolor*^12^, *Triticum aestivum*^13^, and *Oryza sativa*^4^. Plants under stress condition can exhibit signs of tissue dehydration, confirmed by a reduction in their root moisture content^14^. This was reported in *Rosmarinus officinalis* by Sánchez-Blanco et al.^15^ and in *Nerium oleander* by Bañón et al.^16^.

Sap-sucking insects severely decreases potato production worldwide and are important economic pests of crops^17^. Globally, potato farmers consider aphids to be of greater economic importance than defoliators or tuber pests^18^. The green peach aphid, *Myzus persicae* (Sulzer) is a common aphid species that attacks close to 400 crops from diverse families as secondary hosts^19^. These aphids cause major damage to potato plants through feeding, honeydew production, and transmission of viruses^20^. It is reported that severity of drought-stress could cause outbreaks of insect pests^21^. Koricheva et al*.*^22^, in a review, reported insect response to induced water-deficit in woody plants, indicated that the population of sap-sucking insects increases more rapidly on drought-stressed plants than on well-watered plants. However, Huberty and Denno^23^ who worked on drought-stress and its impacts on herbivorous insects reported contrasting results.

Potato (*Solanum tuberosum* L.) is an important global food source^24,25^. Potato plants have comparatively shallow root systems and are sensitive to stress conditions^21^. The impact of drought is a major factor affecting the sustainable production of the crop^26^ Climate change has increased the incidence of irregular weather patterns, comprising low and erratic rainfall patterns that can causes drought and increases pest populations, thereby adversely affecting crop production^27,28^. Thus, the development of potato varieties with high yield, improved quality, and drought and pest resistance is imperative. The use of aphid-resistant potato cultivars has also been proposed as one of the most important strategies for aphid control in China^25,28^.

In our previous research, we found that drought-tolerant potato cultivar was more susceptible to green peach aphids compared to drought-sensitive cultivar^29^. However, the root architectural and biochemical reactions of potato plants to drought-stress and green peach aphid has not been reported. The current research was therefore based on the hypothesis that the responses of root tolerance to drought stress in potato cultivars vary from the responses of root tolerance to aphid infestation. Our specific goal was to determine the root system architecture and biochemical reactions of three potato cultivars to drought stress and green peach aphid infestation under greenhouse conditions. This research will provide a scientific basis for breeding cultivars adapted to regions with frequent drought stress and aphid infestation.

## Results

### Aphid performance

**Figure 1:**
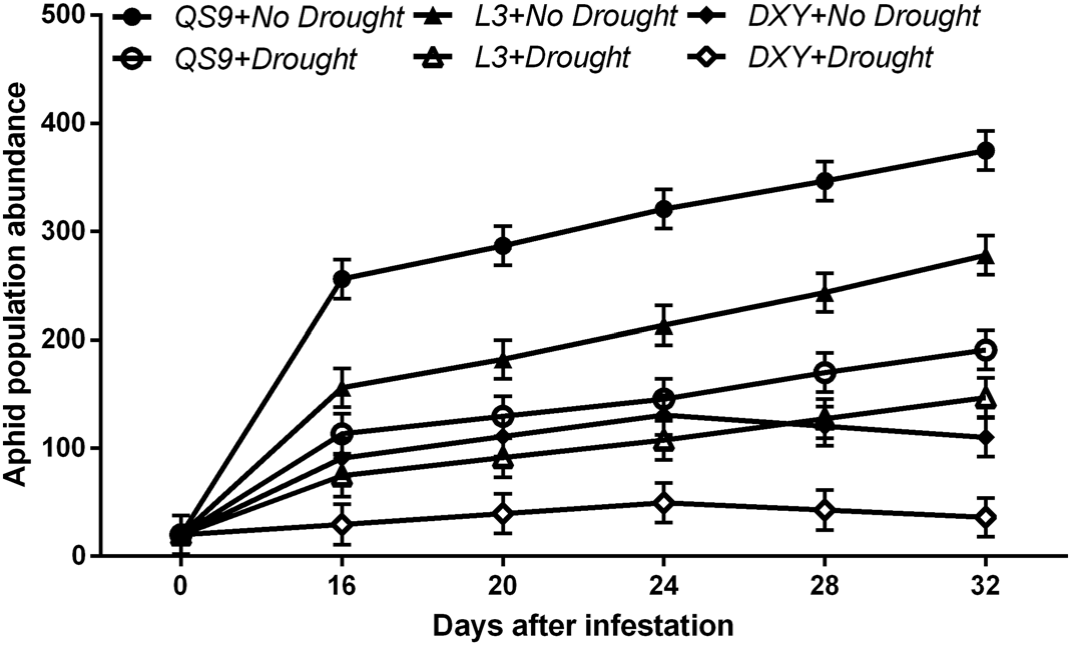
Changes in aphid population abundance in three potato genotypes under two levels of water availability and two levels of aphid infestation. Data represent the mean ± SD of three replicates.

**Figure 2:**
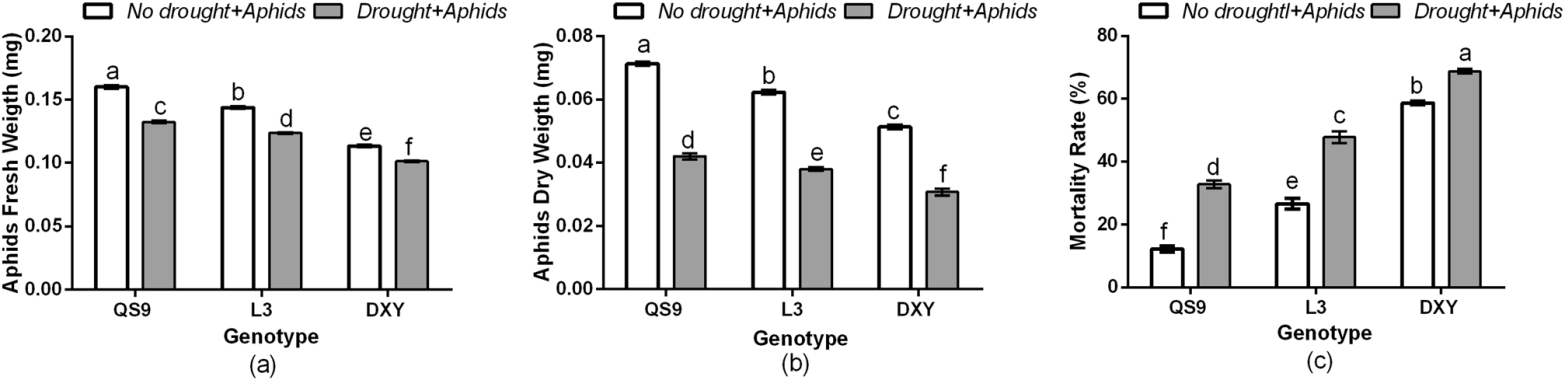
Aphid fresh weight (**a**), dry weight (**b**), and mortality rate (**c**) in three potato genotypes under two levels of water availability and two levels of aphid infestation. Data represent the mean ± SD of three replicates. Lowercase letters indicate means that are significantly different according to the LSD test (*P* < 0.05).

The results showed a significant (*P* < 0.01) variety × drought interaction effect on aphid population abundance. Relative to the initial population, the number of green peach aphids at 28 days post-infestation associated with Qingshu 9, Longshu 3, and Atlantic was increased by 71.2, 68.7, and 43.3%, respectively, under well-watered conditions, and by 64.2, 60.2, and 35.8% under drought stress (Fig. 1). Moreover, green peach aphids reared on Atlantic plants exhibited a 53.1 and 44.4% decrease in population compared with those reared on Qingshu 9 and Longshu 3 plants, respectively, under well-watered and drought-stressed conditions, at 32 days post-infestation. There was also a significant (*P* < 0.01) variety × drought interaction effect on aphid fresh weight, dry weight, and mortality rate (Fig. 2). Drought stress significantly decreased the fresh weight of aphids reared on Qingshu 9, Longshu 3, and Atlantic by 32.7, 36.1, and 39.4% in comparison to the respective controls (Fig. 2a). Drought stress also decreased aphid dry weight of Qingshu 9, Longshu 3, and Atlantic by 48.8, 49.7, and 50.9% in comparison to the control plants (Fig. 2b). Generally, aphid mortality rate was higher on drought-stressed plants. Aphid mortality rate on drought-free (58.6%) and those under drought (68.7%) were highest on Atlantic and least on Qingshu 9 (12.2 and 32.7%, respectively) (Fig. 2c). This suggests that Atlantic and Qingshu 9 were considered the most resistant and susceptible cultivars to aphids, respectively.

### Effect of drought stress and aphid infestation on root conductivity and distribution

**Figure 3:**
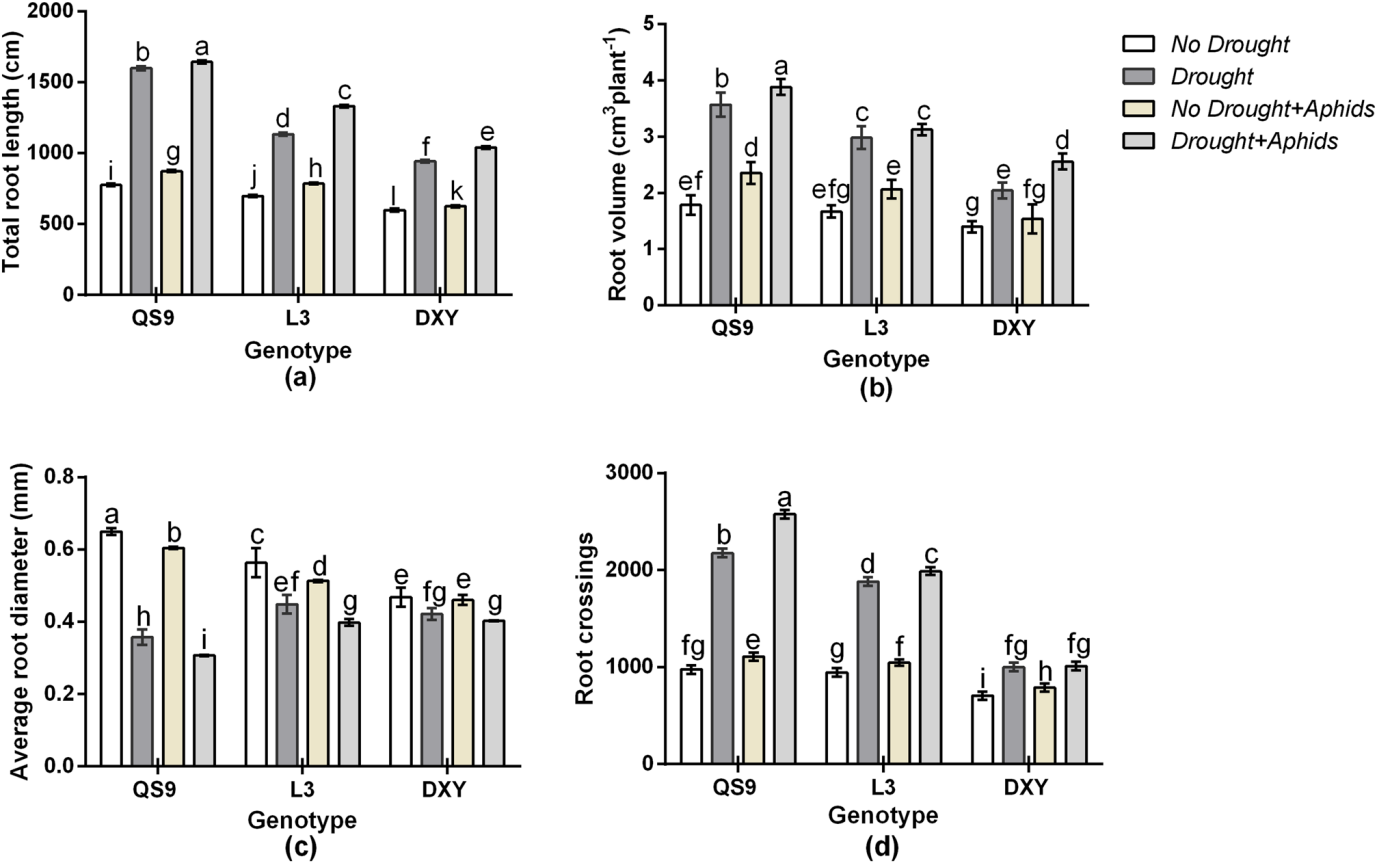
Total root length (**a**), root volume (**b**), average root diameter (**c**), and root crossings (**d**) of three potato genotypes under two levels of water availability and two levels of aphid infestation. Data represent the mean ± SD of three replicates. Lower case letters indicate statistically significant differences between cultivars within the same water treatment and aphid treatment by LSD test (*P* < 0.05).

There was a significant (*P* < 0.02) variety × drought × aphid interaction effect on root crossings. However, total root length, root volume, and average root diameter were not affected (*P* = 0.06). Drought stress increased total root length of Qingshu 9, Longshu 3, and Atlantic by 75.7, 58.7, and 38.5%, respectively, in respect to the control plants. Under aphid infestation, total root length of Qingshu 9, Longshu 3, and Atlantic also increased by 43.4, 31.4, and 21.7% relative to the control plants. Drought and aphid infestation increased total root length of Qingshu 9, Longshu 3, and Atlantic by 79.4, 60.3, and 43.2%, respectively, over the corresponding control plants. The least increase in total root length under drought and aphid stress occurred on Atlantic (Fig. 3a). Drought stress increased root volume of Qingshu 9, Longshu 3, and Atlantic by 50.7, 45.8, and 20.4% respectively, in respect to the control plants. Under aphid infestation, root length of Qingshu 9, Longshu 3, and Atlantic also increased by 38.3, 29.8, and 23.3%, in comparison to the control plants. Drought and aphid infestation increased root volume of Qingshu 9, Longshu 3, and Atlantic by 59.9, 50.1, and 30.7% respectively, more than their control plants (Fig. 3b). Drought stress decreased average root diameter of Qingshu 9, Longshu 3, and Atlantic by 56.4, 19.6, and 15.3%, respectively, in respect to the control plants. Under aphid infestation, average root diameter of Qingshu 9, Longshu 3, and Atlantic also decreased by 15.5, 13.2, and 10.6%, in respect to the control plants. Drought and aphid infestation decreased average root diameter of Qingshu 9, Longshu 3, and Atlantic by 49.9, 16.8, and 12.3%, respectively, in respect to the control plants (Fig. 3c). Under drought stress, root crossings of Qingshu 9, Longshu 3, and Atlantic increased by 79.3, 58.2, and 29.6%, respectively, in respect to the control plants. Under aphid infestation, root crossing of Qingshu 9, Longshu 3, and Atlantic also increased by 17.2, 14.8, and 11.9%, in respect to the control plants. Under drought and aphid infestation, root crossings of Qingshu 9, Longshu 3, and Atlantic increased by 84.6, 60.1, and 30.4%, respectively, compared with the control plants (Fig. 3d). Comparatively, Atlantic had the least total root length, root volume, and root crossings, and the largest average root diameter under both drought stress and aphid infestation. The greatest total root length, root volume, and root crossings, and the least average root diameter among the cultivars occurred on Qingshu 9 (Fig. 3).

### Effect of drought stress and aphid infestation on root proliferation

**Figure 4:**
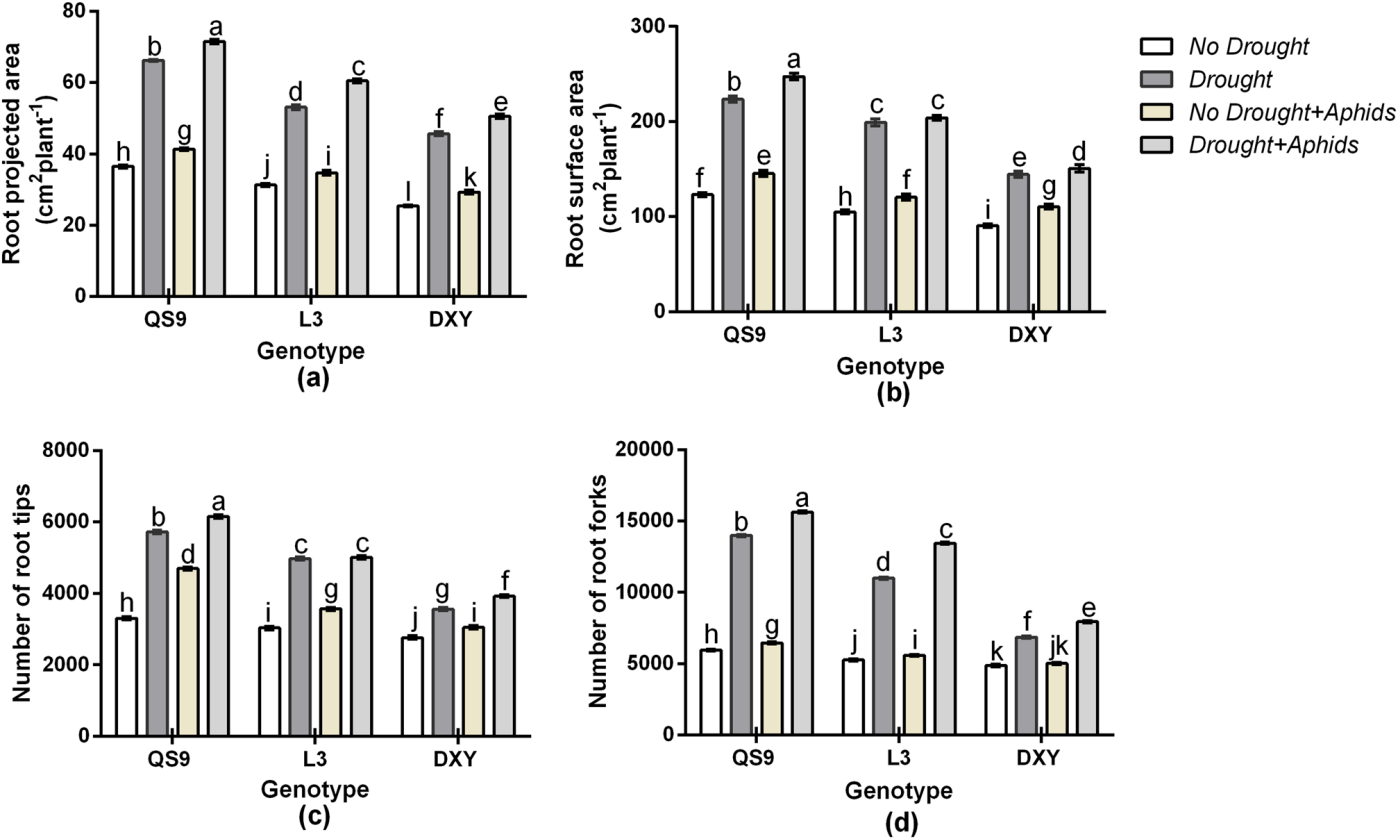
Root projected area (**a**), root surface area (**b**), number of root tips (**c**), and number of root forks (**d**) of three potato genotypes under two levels of water availability and two levels of aphid infestation. Data represent the mean ± SD of three replicates. Lower case letters indicate statistically significant differences between cultivars within the same water treatment and aphid treatment by LSD test (*P* < 0.05).

**Figure 5:**
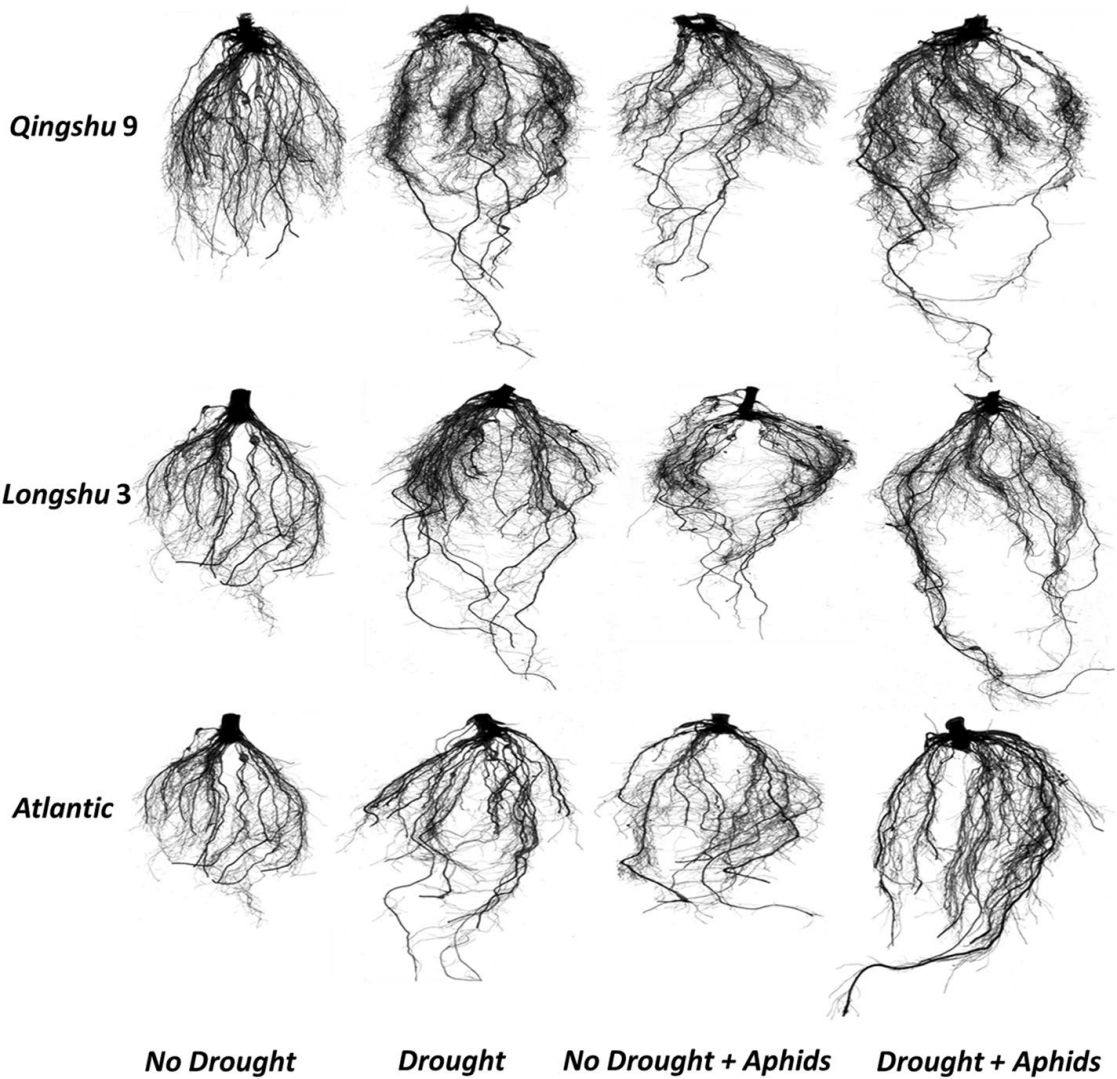
Scan images of the root system of three potato genotypes at 60 days after drought and aphid infestation treatments. Digital images of the root system were produced with a root scanner (STD 4800, EPSON, Quebec City, Canada).

Drought stress and aphid infestation caused an increase in root projected area, root surface area, and number of root tips, of all cultivars relative to their corresponding control plants (Fig. 4). Aphid infestation marginally increased the number of root forks of Qingshu 9 and Longshu 3 plants, but did not affect Atlantic plants irrespective of aphid attack. Drought stress increased root projected area of Qingshu 9, Longshu 3, and Atlantic by 58.3, 47.4, and 31.5%, respectively, in respect to the control plants. Under aphid infestation, root projected area of Qingshu 9, Longshu 3, and Atlantic also increased by 19.3, 16.1, and 10.7%, relative to the control plants. Drought and aphid infestation increased root projected area of Qingshu 9, Longshu 3, and Atlantic by 64.6, 54.1, and 41.3%, respectively, in respect to the control plants (Fig. 4a). Under drought stress, the root surface area of Qingshu 9, Longshu 3, and Atlantic increased by 52.6, 49.6, and 25.1%, respectively, in respect to the control plants. Under aphid infestation, root surface area of Qingshu 9, Longshu 3, and Atlantic also increased by 19.4, 15.6, and 12.5%, relative to the control plants. Under drought and aphid infestation, the root surface area of Qingshu 9, Longshu 3, and Atlantic increased by 57.8, 51.1, and 29.5%, respectively, in respect to the control plants (Fig. 4b). Drought stress increased number of root tips of Qingshu 9, Longshu 3, and Atlantic by 49.9, 39.5, and 19.7%, respectively, in respect to the control plants. Under aphid infestation, number of root tips of Qingshu 9, Longshu 3, and Atlantic also increased by 33.6, 20.2, and 16.9%, relative to the control plants. Drought and aphid infestation increased number of root tips of Qingshu 9, Longshu 3, and Atlantic by 53.8, 41.1, and 24.9%, respectively, in respect to the control plants (Fig. 4c). Drought stress significantly (*P* < 0.01) increased number of root forks of Qingshu 9, Longshu 3, and Atlantic by 58.3, 50.4, and 27.9%, respectively, in respect to the control plants. Under aphid infestation, number of root tips of Qingshu 9, Longshu 3, and Atlantic also increased by 16.9, 11.8, and 9.7%, relative to the control plants. Drought and aphid infestation increased number of root forks of Qingshu 9, Longshu 3, and Atlantic by 61.4, 59.5, and 49.9%, respectively, in respect to the control plants (Fig. 4d). Comparatively, the least root projected area, root surface area, number of root tips, and number of root forks occurred on Atlantic under both drought stress and aphid infestation. The greatest root projected area, root surface area, number of root tips, and number of root forks among cultivars occurred on Qingshu 9 plants (Fig. 4d). Drought and aphid stress effect caused an increase in the sizes of the root systems of all the cultivars (Fig. 5).

### Effect of drought stress and aphid infestation root dehydration

**Figure 6:**
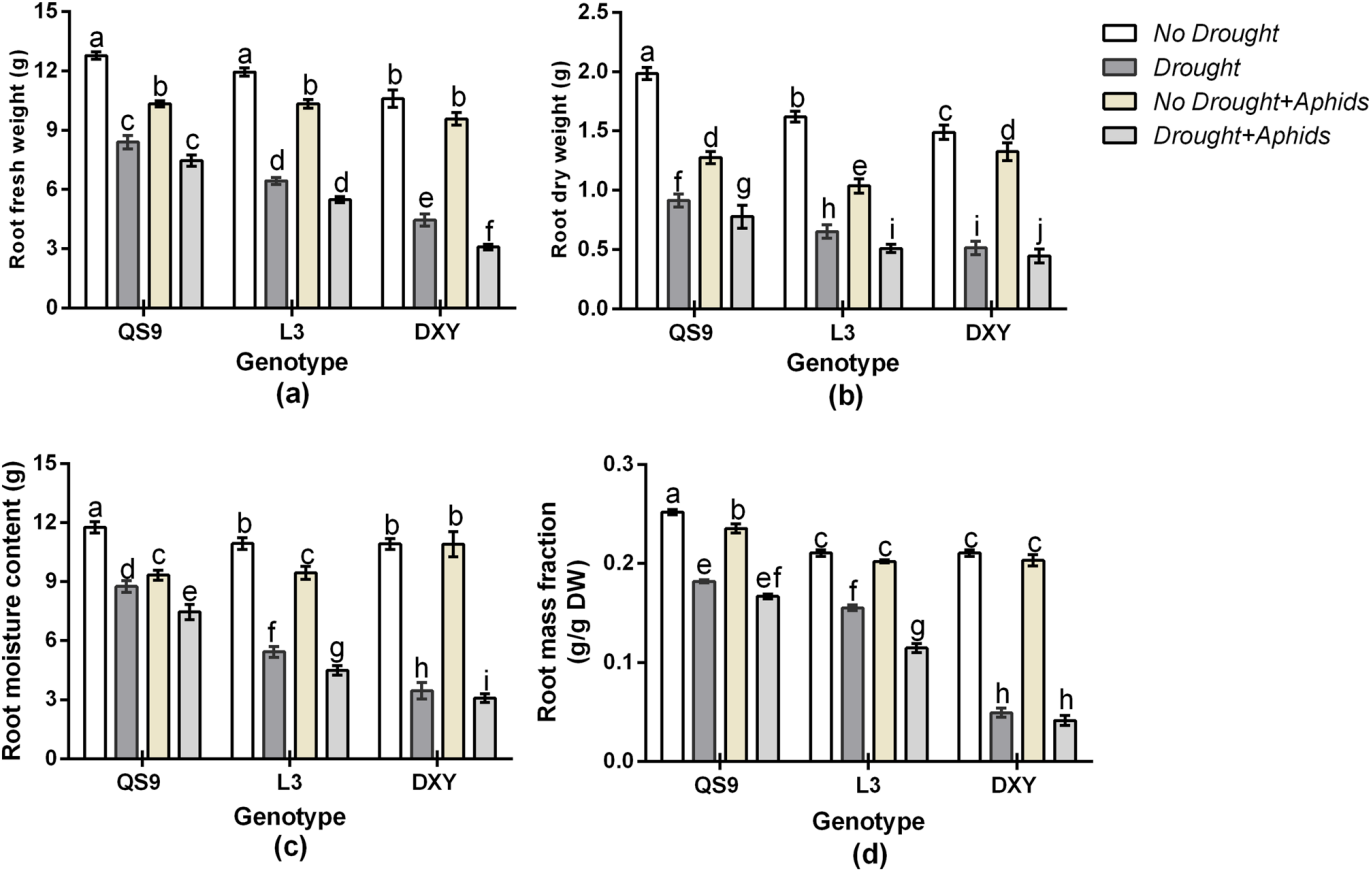
Root fresh weight (**a**), dry weight (**b**), moisture content (**c**), and mass fraction (**d**), of three potato genotypes under two levels of water availability and two levels of aphid infestation. Data represent the mean ± SD error of three replicates. Lower case letters indicate statistically significant differences between cultivars within the same water treatment and aphid treatment by LSD test (*P* < 0.05).

Root fresh weight, dry weight, moisture content, and mass fraction differed among cultivars under the control condition (Fig. 6). Although aphid infestation slightly decreased root fresh weight, moisture content, and mass fraction of Qingshu 9 and Longshu 3 plants, Atlantic plants were not affected. Drought stress decreased root fresh weight of Qingshu 9, Longshu 3, and Atlantic by 42.4, 58.4, and 65.7%, respectively, in respect to the control plants. Under aphid infestation, root fresh weight of Qingshu 9, Longshu 3, and Atlantic also decreased by 36.3, 32.4, and 19.1%, relative to the control plants. Drought and aphid infestation decreased root fresh weight of Qingshu 9, Longshu 3, and Atlantic by 45.5, 60.2, and 69.8%, respectively, in respect to the control plants (Fig. 6a). Drought stress decreased root dry weight of Qingshu 9, Longshu 3, and Atlantic by 34.6, 51.2, and 59.9%, respectively, in respect to the control plants. Under aphid infestation, root dry weight also decreased by 40.5, 31.4, and 17.2%, relative to the control plants (Fig. 6b). Drought and aphid infestation decreased root dry weight of Qingshu 9, Longshu 3, and Atlantic by 41.5, 61.1, and 62.1%, respectively, in respect to the control plants. Under drought stress, root moisture content of Qingshu 9, Longshu 3, and Atlantic significantly (*P* < 0.01) decreased by 21.1, 55.7, and 76.3%, respectively, in respect to the control plants. Under aphid infestation, root moisture content of Qingshu 9, Longshu 3, and Atlantic also decreased by 20.7, 17.6, and 11.2%, relative to the control plants. Under drought and aphid infestation, root moisture content of Qingshu 9, Longshu 3, and Atlantic decreased by 30.2, 59.9 and 79.8%, respectively, in respect to the control plants (Fig. 6c). Drought stress decreased root mass fraction of Qingshu 9, Longshu 3, and Atlantic by 44.6, 54.4, and 61.1%, respectively, in respect to the control plants. Under aphid infestation, root mass fraction of Qingshu 9, Longshu 3, and Atlantic also decreased by 19.5, 13.4, and 8.3%, relative to the control plants. Drought and aphid infestation decreased root mass fraction of Qingshu 9, Longshu 3, and Atlantic by 47.5, 59.8, and 63.3%, respectively, in respect to the control plants (Fig. 6d). Comparatively, Atlantic had the highest decrease of root fresh weight, root dry weight, root moisture content, and root mass fraction under drought stress. Nonetheless, the least decrease of root fresh weight, root dry weight, root moisture content, and root mass fraction of the cultivars occurred on Qingshu 9 plants. In contrast, under aphid infestation, the greatest decrease of root fresh weight, root dry weight, root moisture content, and root mass fraction under drought stress occurred on Qingshu 9, whereas the least decrease occurred on Atlantic (Fig. 6).

### Root biomass response to drought stress and aphid infestation

**Figure 7:**
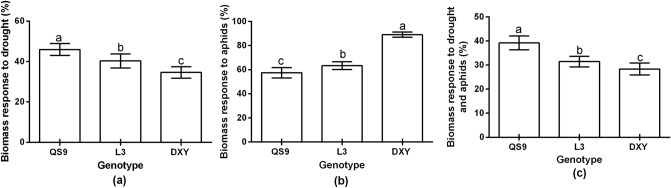
Biomass response to drought (**a**), aphids (**b**), and drought and aphids (**c**) in comparison to the control for three potato genotypes under two levels of water availability and two levels of aphid infestation. Data represent the mean ± SD of three replicates. Lowercase letters indicate means that show statistically significant differences between treatments according to the LSD test (*P* < 0.05).

Drought and aphids stress significantly (*P* < 0.01) influenced root biomass accumulation of the potato cultivars. The highest biomass accumulation (45.9%) occurred on the Qingshu 9 cultivar under drought stress (Fig. 7a). However, under aphid infestation, the highest biomass occurred on Atlantic plants (Fig. 7b). Moreover, under the effect of both drought and aphid stress, Qingshu 9 had the highest (39.1%) biomass accumulation (Fig. 7c). Plant root biomass was greater in Atlantic (89.1%), but lower in Qingshu 9 (57.5%), under aphid stress. Accordingly, the Atlantic and Qingshu 9 cultivars were considered the most tolerant and susceptible cultivars to aphids, respectively.

### Effect of drought stress and aphid infestation on MDA, H_2_O_2_, and Pro contents

**Figure 8:**
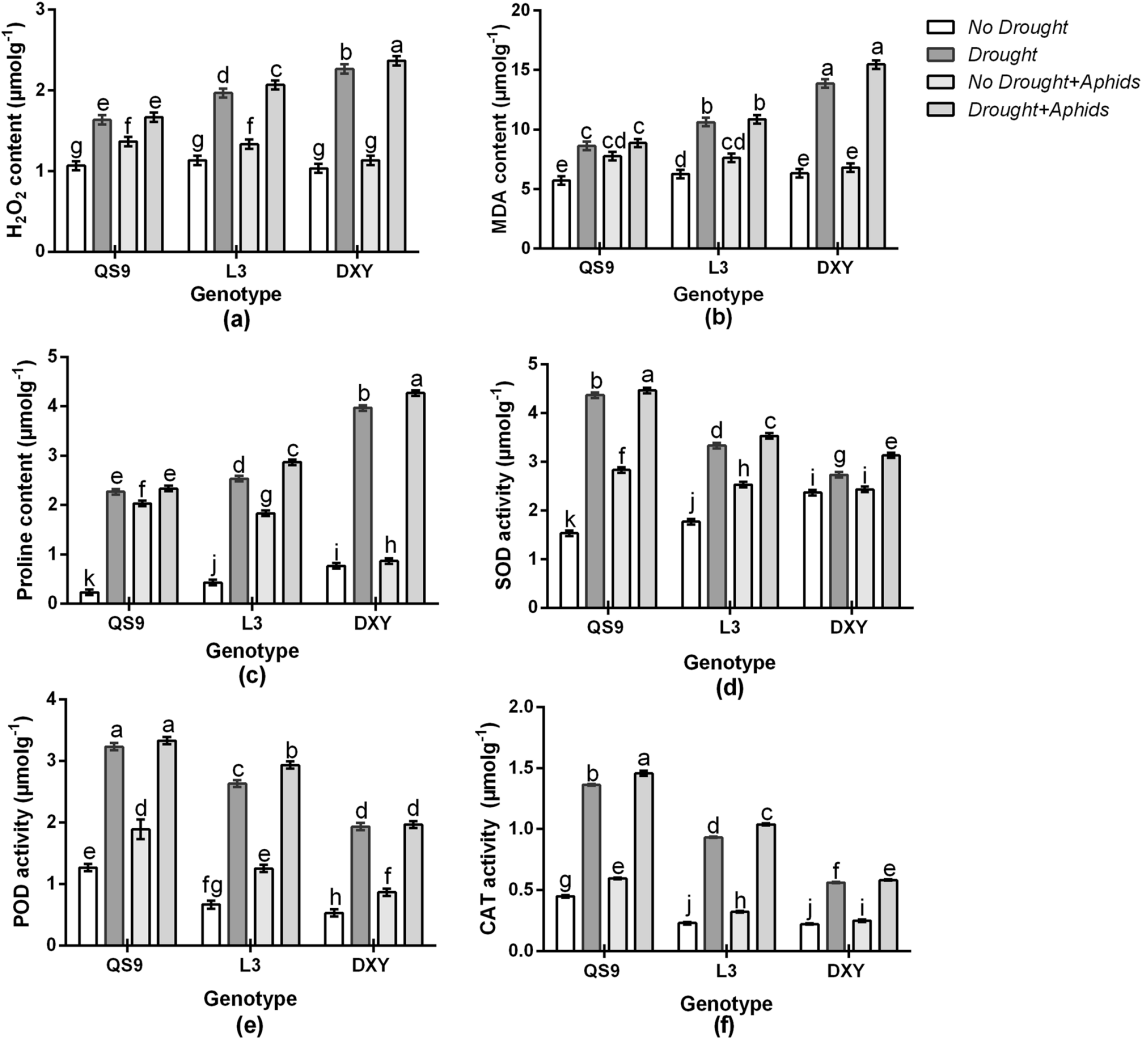
H_2_O_2_ content (**a**), MDA content (**b**), Pro content (**c**), SOD activity (**d**), POD activity (**e**), and CAT activity (**f**) of three potato genotypes under two levels of water availability and two levels of aphid infestation. Data represent the mean ± SD of three replicates. Lower case letters indicate statistically significant differences between cultivars within the same water treatment and aphid treatment by LSD test (*P* < 0.05).

To associate the functional attributes of H_2_O_2_, MDA, and Pro with drought and aphid tolerance exhibited by some of the cultivars, MDA, H_2_O_2_ and Pro contents were evaluated. Consequently, H_2_O_2_, MDA, and Pro contents differed among cultivars under the control condition (Fig. 8a-c). Drought stress increased H_2_O_2_ content of Qingshu 9, Longshu 3, and Atlantic by 43.6, 57.6, and 78.4%, respectively, in respect to the control plants. Under aphid infestation, H_2_O_2_ content of Qingshu 9, Longshu 3, and Atlantic also increased by 29.5, 19.4, and 10.1%, relative to the control plants. Drought and aphid infestation increased H_2_O_2_ content of Qingshu 9, Longshu 3, and Atlantic by 45.1, 60.3, and 80.2%, respectively, in respect to the control plants. Drought stress increased MDA content of Qingshu 9, Longshu 3, and Atlantic by 30.9, 44.8, and 53.2%, respectively, in respect to the control plants. Under aphid infestation, MDA content of Qingshu 9, Longshu 3, and Atlantic also increased by 29.1, 20.4, and 14.3%, relative to the control plants. Drought and aphid infestation increased MDA content of Qingshu 9, Longshu 3, and Atlantic by 32.7, 46.9, and 56.6%, respectively, in respect to the control plants. Drought stress increased Pro content of Qingshu 9, Longshu 3, and Atlantic by 75.5, 80.4, and 89.8%, respectively, in respect to the control plants. Under aphid infestation, Pro content also increased by 69.3, 65.1, and 21.6%, reletive to the control plants. Drought and aphid infestation increased Pro content of Qingshu 9, Longshu 3, and Atlantic by 77.1, 84.9, and 90.9%, respectively, in respect to the control plants. Comparetively, the greatest MDA, H_2_O_2_, and Pro contents occurred in Atlantic plants, under drought stress, whereas the lowest values were recorded in Qingshu 9 plants. In contrast, the greatest contents of MDA, H_2_O_2_, and Pro occurred in Qingshu 9 plants under aphids stress.

### Effect of drought stress and aphid infestation on antioxidant enzyme activities

To evaluate whether variances can be related with the cultivars’ resistance to drought and aphid stress, the changes in the scavenging activity of ROS such as SOD, POD, and CAT were examined. The results indicate that SOD, POD, and CAT activities increased in all cultivars under drought stress and aphid infestation compared to their corresponding controls (Fig. 8d-f). Under drought stress, SOD activity in Qingshu 9, Longshu 3, and Atlantic increased by 75.5, 51.7, and 24.1%, respectively, in respect to control plants. Under aphid infestation, SOD activity in Qingshu 9, Longshu 3, and Atlantic also increased by 51.4, 30.3, and 13.5%, relative to control plants. Under drought and aphid infestation, SOD activity in Qingshu 9, Longshu 3, and Atlantic increased by 79.7, 58.9, and 34.2%, respectively, in respect to control plants. Under drought stress, POD activity in Qingshu 9, Longshu 3, and Atlantic increased by 61.5, 55.8, and 47.5%, respectively, in respect to the control plants. Under aphid stress, POD activity in Qingshu 9, Longshu 3, and Atlantic also increased by 35.4, 34.1, and 20.5%, relative to control plants. Under drought and aphid infestation, POD activity in Qingshu 9, Longshu 3, and Atlantic increased by 64.1, 59.9, and 54.1%, respectively, in respect to the control plants. Drought stress increased CAT in Qingshu 9, Longshu 3, and Atlantic by 54.3, 49.2, and 29.6%, respectively, in respect to control plants. Under aphid infestation, CAT activity in Qingshu 9, Longshu 3, and Atlantic also by 38.2, 30.8, and 16.9%, relative to control plants. Drought and aphids infestation increased CAT in Qingshu 9, Longshu 3, and Atlantic by 59.8, 52.7, and 38.9%, respectively, in respect to control plants. Comparatively, higher SOD, POD, and CAT activities occurred in Qingshu 9 plants than the other cultivars, both under drought and aphid stress, while the least SOD, POD, and CAT activities occurred in Atlantic plants.

## Discussion

The responses of aphids to drought stress reported in previous studies have indicated contrasting findings^30,31^. Koricheva et al*.*^22^ reported that aphid population increased on drought-stressed cultivars than on well-watered cultivars, whereas Huberty and Denno^23^ found decreases in their population under similar condition. Few field trials support the notion that aphid population increases on drought-stressed cultivars; experimentally imposed water deficit, nonetheless, often negatively influences aphid population abundance^23^. In this study, aphid population was higher on the drought-free plants compared with the drought-stressed plants across all cultivars. Drought stress negatively affected aphid population, which led to greater mortality rate and decreased biomass of the aphids in all the cultivars. Other studies have produced contradictory or varying results^32,33^. However, the response of insect pest population to drought stress is suggested to hinge on the variety of plant and the intensity of stress^34^. An experiment on *Brassica oleracea* showed that aphid population increased on drought-free plants compared with the drought-stressed plants^35^. Moreover, Floater^36^ recorded greater aphid mortality and lower aphid biomass on drought-stressed plants. In the present study, the drought-sensitive cultivar showed more resistance to aphid infestation under both water treatments. This cultivar also exhibited low aphid population abundance and a high aphid mortality rate. Conversely, the drought-tolerant cultivar was susceptible to the aphids and showed greater aphid population abundance and a low aphid mortality rate. It appears that aphid performance correlates positively with high water content in the host plant, under drought condition. The loss of water of all cultivars under drought stress increased mortality rate and decreased the fresh weight of aphids. Moreover, the tolerant cultivar (Qingshu 9) exhibited wide variation in root hydraulic conductivity traits, confirmed by increasing root fresh weight, root dry weight, root moisture content, root mass fraction, and root biomass, which led to greater availability of sap for the aphids to feed on. Accordingly, Qingshu 9 was the most susceptible host because the aphids survived better on it. In contrast, the sensitive cultivar (Atlantic) that possesses poor root hydraulic conductivity, exhibited signs of tissue dehydration, confirmed by a reduction in root fresh weight, root dry weight, root moisture content, root mass fraction, and root biomass that possibly starved the aphids to death over time. Noticeably, the moderately tolerant cultivar, Longshu 3, was not extremely susceptible, neither was it extremely resistance to the green peach aphid. However, comparatively, the green peach aphid performed better on it than the Atlantic cultivar, under drought stress. This was confirmed by the high aphid population abundance and low aphid mortality rate, compared with the sensitive cultivar. This is probably due to its ability to maintain turgor pressure by improving its hydraulic conductivity, compared with the Atlantic cultivar. The Longshu cultivar can also be utilize in areas where both drought and aphids are major concern. It is reported that, variations in host plant physical and chemical composition can have important consequences on herbivore population dynamics^37^. Potato varieties differ in the volatile profiles in their headspace and these differences elicit different behavior from the green peach aphid^38^. Although the high dehydration of Atlantic plants greatly increased aphids mortality rate under drought condition, Atlantic also exhibited higher resistance to aphids under well-watered condition. This suggest that the drought-sensitive cultivar may contain secondary metabolites that act as repellants to the peach aphid. We therefore speculate that plants natural defense against aphid attack and water availability contributes significantly to the outcome of aphid population abundance. Thus, host plant response to stress condition should be assessed when considering the response of herbivore insects to drought stress. The Atlantic cultivar can be utilized to protect against losses in potato production in regions where peach aphids are a key pest of potato as proposed by Xu et al.^39^.

Plants with better root conductivity and distribution are able to thrive well under drought stress due to their ability to source water^40^. The roots of stressed plants tend to increase and spread into deeper soil layers to obtain resources^41^. The present results show that drought and aphids stress increased total root length, root volume, and root crossings of all cultivars relative to their corresponding control plants. However, the average root diameter decreased under drought and aphid stress across all cultivars. A similar result was reported on *Oryza sativa*^42^, *Cicer arientinum*^43^, and *Sorghum bicolor*^44^*.* The Atlantic cultivar had the least total root length, root volume, and root crossings, and the greatest average root diameter under both drought and aphis stress. However, the greatest total root length, root volume, and root crossings, and the least average root diameter among cultivars occurred on Qingshu 9. Drought-tolerant varieties are known to be capable of increasing their root depth, root volume, and root crossings significantly more than sensitive varieties under stress conditions in legumes^45^. Previous studies reported that plants with thicker roots tend to penetrate deeper under drought stress^46^. These results are in contrast with previous reports that increase in root diameter is a significant trait in tolerant cultivars under water stress conditions. However, it agrees with others who reported that small root diameter support plants to significantly improve hydraulic conductance by increasing the amount of surface area in contact with soil water^47^. These results suggest that total root length, root volume, root crossings, and average root diameter contributed significantly in the Qingshu 9 plants tolerant to drought, but this did not improve the plants resistance to aphids attack.

In order to access available soil moisture under drought stress, plants adapt by greater root proliferation^3^. Root proliferation is generally governed by the initiation and elongation of lateral roots, which usually refers to lateral root number, root projected area, root surface area, number of root tips, and number of root forks^42^. Plants with greater root proliferation have comparatively great water uptake efficiency under stress condition^48^. *Cicer arietinum* lines with greater proliferation have been reported to perform better in yield and drought tolerance related traits under water deficit environments^49^. In the present study, drought stress and aphid infestation caused significant increase in the root projected area, root surface area, number of root tips, and number of root forks of all cultivars, relative to their corresponding control plants. Branching of roots of *Silene vulgaris* was increased under drought stress^47^. Likewise, root surface area of *Silene vulgaris* was increased under drought stress^47^. The least root projected area, root surface area, number of root tips, and number of root forks occurred on Atlantic under both drought and aphid infestation. Nevertheless, the greatest root projected area, root surface area, number of root tips, and number of root forks occurred on Qingshu 9 plants. The benefit of a deep and proliferative root system for tolerant cultivars under stress conditions has been reported in various crops, including *Oryza sativa* (Uga et al*.*^4^), *Zea mays*^5^, *Hordeum vulgare*^6^, *Triticum aestivum*^7^, *Brassica napus*^8^, and *Glycine max*^9^. The Atlantic cultivar, which exhibited poor root system architecture under both drought and aphid infestation, showed greater resistance to aphids with or without drought stress.

In plant cells, H_2_O_2_ is produced through aerobic metabolism, and as a detrimental oxygen derivative, it can cause cellular damage^50^. Greater accumulation of MDA indicates enhanced production of reactive oxygen species^51,52^. One of the early signal measures during stress condition in plants usually includes the accumulation of Pro^53^. Proline acts as an intermediary of osmotic adjustment in plants under stress conditions^52^. Our results showed that, all cultivars under drought and aphid stress accumulated more H_2_O_2_, MDA, and Pro compared to their corresponding control plants. However, Atlantic plants significantly increased H_2_O_2_, MDA, and Pro contents compared to the other cultivars, under drought stress, whereas, under aphid stress, the increase of H_2_O_2_, MDA, and Pro contents was greater in Qingshu 9. The accumulation of H_2_O_2_, MDA, and Pro in Atlantic under aphid infestation did not vary significantly from the control, probably because of this cultivar’s resistance to aphids. The greater accumulation of Pro content in all the drought-stressed plants did not inhibit the population abundance of the green peach aphid, probably because Pro might have function as a stress-related signal^54^.

Antioxidant enzyme protections comprising SOD, POD, and CAT directly scavenge superoxide radicals and H_2_O_2_^55^. Other studies have confirmed that SOD, POD, and CAT activities increased in response to stress in *Glycine max*^56^ and *Panicum virgatum*^57^. Consistent with our results, increased SOD, CAT, and POD activities were observed across all cultivars, under drought and aphid stress. However, the amount of accumulation differed between cultivars. The highest SOD, POD, and CAT activities occurred in Qingshu 9; both under drought and aphid stress, while the least SOD, POD, and CAT activities was observed in Atlantic. The accumulation of antioxidant enzymes in plants is reported as a signal of its level of tolerance to stress^55^. The greater antioxidant enzyme activities observed in Qingshu 9 did not enhance their resistance to the aphid. The least antioxidant enzyme activities occurred in Atlantic, under aphids stress. However, it inhibited the population abundance of the aphid. This was demonstrated by the higher biomass accumulated in Atlantic under aphid stress. This could be due to the inhibitory compounds in Atlantic cultivar. Thus, the results of our study propose that the root mechanism of tolerance to drought among potato plants differ from the mechanism that may enhance aphid resistance.

## Conclusions

The results of this study demonstrate that Atlantic, which possesses a root system sensitive to drought, showed greater resistant to the peach aphid under both water treatments. This cultivar also exhibited poor aphid population abundance, high mortality rate, and higher biomass accumulation under aphid stress. Moreover, the moderately drought tolerant cultivar, Longshu 3, was not extremely susceptible, neither was it extremely resistant to the green peach aphid. However, this cultivar showed high aphid population abundance and low aphid mortality rate, compared with the sensitive cultivar. The Qingshu 9, which possesses root system tolerant to drought, was extremely susceptible to the aphid and demonstrated high aphid population abundance, low aphid mortality rate, and low biomass accumulation under aphid stress. Moreover, dehydration of the Atlantic cultivar decreased the population abundance of the aphid under drought stress. The Qingshu 9 cultivar, which had enhanced root system, probably improved water uptake and led to greater availability of sap on which the aphid survived and increased its population. The resistance of Atlantic to the peach aphid under both water treatments may also be due to the presence of secondary metabolites in the cultivar. Thus, the Atlantic cultivar can be utilized to protect against losses in potato yield in areas where peach aphids are a major pest of potato. The Longshu 3 cultivar can also be utilized in areas where both drought and aphids are major concern. This study indicates that the root architecture and biochemical trait that enhances potato tolerance to drought do not necessarily correlate to a plant’s tolerance to aphids.

## Materials and methods

### Study area

The experiment was conducted at Gansu Agricultural University in Lanzhou, Gansu, China (36°02 N, 104°25 E; 2400 m a.s.l.). The annual mean temperature of Lanzhou is 6.5 °C (a maximum of 19.0 °C in July and a minimum of − 8.0 °C in January) and the annual precipitation is 395 mm.

### Growth conditions and plant material

Growth conditions and plant materials were used as described in our previous study^29^. Briefly, the experiment was conducted in a greenhouse (day temperature 25–35 °C, night temperature 18–22 °C, daytime relative humidity 45–55%, light intensity 15,000–18,000 lx) during the summer of 2019. This experiment utilized three potato cultivars of inflorescence emergence maturity. Mini tubers of the potato cultivars were acquired from Gansu Haofeng Seed Company Limited, Lanzhou, China for the experiment. The experiment was conducted on three potato cultivar, which vary in tolerance to drought: Qingshu 9 (QS9; tolerant)*,* Longshu 3 (L3; moderately tolerant) and Atlantic (DXY; sensitive). One tuber per pot was sown per plastic pots (12.5 cm diameter, 9.5 cm deep) filled with 2 kg of loamy soil. Drought was imposed by withholding watering at 40 days after sowing and allowing the moisture content of the soil to drop to 30% soil capacity. The moisture contents of the various treatments were monitored daily and adjusted as and when necessary, by adding the required amount of water.

### Aphid culture

Adults of *Myzus persicae* were collected from potato plants at the Experimental Farm of Gansu Agricultural University in Lanzhou, China. These aphids were reared on potato plants (Longshu 10) in ventilated glass cages. The culture was maintained in an a controlled environment room at 19 ± 1 °C under a 16:8 h light:dark photo cycle and relative humidity 55%. The culture was preserved for 6 months before using for the trial.

### Experimental design and treatments

A 3 × 2 × 2 factorial experiment in a split-split plot design with three replications was conducted in a greenhouse. The treatments were: three potato cultivars, Qingshu 9 (QS 9), Longshu 3 (L 3), and Atlantic (DXY), two levels of water availability to plants (well-watered and drought-stressed), and two levels of aphid infestation (aphid infestation and no aphid infestation). The three potato cultivars were allocated to the main plots. The main plots were split into sub plots for the drought-stress and well-watered treatments. The split-plots were further split for the allocation of the aphid and no aphid treatments. Six pots per experimental unit were assigned to a water treatment. A total of 216 pots were used for the experiment. In each experimental unit, six plants were sampled for data collection, giving a total of six subsamples in each of the three replications for each treatment. The well-watered plants were defined as plants growing in soil with a water content of 100% of soil capacity, whereas the drought-stressed plants were defined as plants growing in a soil with a water content of 30% of soil capacity^58^. Soil capacity was determined by applying known volume of water per pot and allowing the excess water to drain through the perforated holes at the base of the plastic pot. The excess water was collected until no more water drained out. The amount of water collected was subtracted from the amount of water applied and the difference was considered the amount of water for field capacity. Prior to exposing the various cultivars to drought and aphid treatments, all the pots were maintained at field capacity by regular watering. The well-watered treatments were watered regularly to maintain the field capacity (100% soil moisture) throughout the experiment. For the ‘Drought plants’, watering was withheld and monitored with soil moisture meter (Delta-T Devices, Cambridge, UK) until the water level dropped to 30%. To maintain the levels of water through time in each treatment, Delta-T Theta Probe ML2 (Delta-T Devices, Cambridge, UK) was used to measure the soil moisture content and a little amounts of water was applied to the ‘Drought plants’ to maintain the 30% soil capacity in each of the pots containing the drought treated plants. This procedure was repeated until the end of the experiment.

### Determination of aphid population abundance, biomass, mortality rate, and tolerance index

Sixty day old potato plants were infested with aphid nymphs and monitored for 32 days. Six shoots of each cultivar in each three replications were infested with 20 nymph aphids for both levels of water availability to treatments. The aphids were introduced to the designated plants at 20 days after drought treatment. Each of the shoots was completely enclosed separately with a nylon mesh cage to prevent aphid escape. Aphid nymphs were allowed to develop into adults and reproduce on each plant for 32 days. Aphid numbers on each plant were determined on day 16, 20, 24, 28, and 32. Adults from each plant were pooled and weighed immediately after they were collected, dried at 60 °C for 24 h, and then weighed again. Aphid mortality rate was measured by counting the number of dead aphids, together with counts of their nymphs (including live and dead nymphs) on each plant. Aphid mortality rate (%) was then calculated as:

1 $$ {\text{AMR}} = \frac{{\text{Number of dead aphis}}}{{\text{Total number of nymphs}}} \times 100 $$

### Measurement of root indexes

The roots of 16 plants were collected at the end of the experiment to examine the root indexes. Roots of the sampled plants (16 plants per treatment) were washed in distilled water and cut from the shoots. To obtain a digital image of the root system, roots of individual plants were scanned with a root scanner (STD 4800, EPSON, Quebec City, Canada). Total root length (TRL), root volume (RV), root surface area (RSA), root projected area (RPA), number of root tips (NRT), number of root forks (NRF), average root diameter (ARD), and root crossings (RC) per plant were determined using the root image analysis software, Win RHIZO version 5.0 (Regent Instruments, Inc., Quebec City, Canada).

### Determination of relative root biomass response to stress (RBRS)

The biomass response to stress (BRS) was calculated from a comparison between the root index under stress and the root index under no stress for each potato variety. The variety with the highest RBRS for most of the measured root indexes was considered the most tolerant among the tested varieties. The RBRS was calculated as:

2 $$ {\text{BRS}} = \frac{{\text{Root biomass under stress}}}{{\text{Root biomass under no stress}}} \times 100 $$

### Root moisture content analysis

The roots of the sampled plants were washed and cut from the shoots and weighed immediately for measurement of fresh weight. Dry weight was determined after roots were dried at 80 °C in an oven for 72 h. Root moisture content (RMC) was calculated as:

3 $$ {\text{RMC}} = {\text{FW}} - {\text{DW}} $$

where FW is fresh weight and DW is dry weight.

### Determination of root dehydration

The root mass fraction was determined as described by Franco et al.^14^. In brief, the roots of A total of 16 plants of each treatment were collected at the end of the experiment. Plant materials were dried in an oven to constant weight at 80 °C for 72 h to record total dry weight after determining their fresh weight.

### Proline (Pro) content analysis in root samples

Proline content in root samples was determined according to the method described previously^59^. Root samples were homogenized in 3% 5-sulfosalicylic acid solution after extraction at room temperature. Proline content was determined using a standard curve and free Pro content was expressed as μmol g^−1^ fresh weight of root.

### Hydrogen peroxide (H_2_O_2_) content analysis in root samples

Hydrogen peroxide levels in root samples were determined as described previously by Mostofa et al.^60^. Root samples (0.5 g) were homogenized in an ice bath with 5 mL 0.1% (w/v) trichloroacetic acid (TCA). The homogenate was centrifuged at 12,000 g for 20 min. The supernatant (0.5 mL) was mixed with 0.5 mL 10 mM potassium phosphate (K_3_PO_4_) buffer (pH = 7.0) and 1 mL 1 MKI. The absorption of the supernatant was measured at 390 nm, and the content of H_2_O_2_ was calculated using the H_2_O_2_ reference standard curve.

### Malondialdehyde (MDA) content analysis in root samples

Malondialdehyde in root samples was determined according to the method of Liu et al.^61^. In brief, root samples (0.1 g) were homogenized in 5% (w/v) TCA. The homogenate was centrifuged at 10,000 g for 5 min. The supernatant (0.5 mL) was mixed with 1 mL of 0.5% (w/v) TBA in 20% TCA. The supernatant was used for MDA assay.

### Antioxidant enzyme activities analysis in root tissues

About (0.5 g) of root tissue was ground with liquid nitrogen and the total root protein was extracted with 0.05 M potassium phosphate (pH = 7.0). After centrifugation at 12,000 g for 15 min at 4 °C, the supernatant was used to determine superoxide dismutase (SOD, EC 1.15.1.1), catalase (CAT, EC 1.11.1.6) and peroxidase (POD, EC 1.11.1.7) activities. The SOD activity was determined as described by Giannopolitis and Ries^62^. The POD activity was measured at 470 nm as described by Chance and Maehly^63^, and the CAT activity was estimated according to the method described by Nakano and Asada^64^.

### Statistical analysis

Statistical analysis was performed using SPSS statistics software (Version 19.0, SPSS, Chicago, IL, USA Lower case letters indicate statistically significant differences between cultivars within the same water treatment and aphid treatment by LSD test (*P* < 0.05). The results are presented as means ± SD.
